# Supplementary material for: Multi‐dataset identification of innovative feature genes and molecular mechanisms in keratoconus
Source: J Cell Mol Med. 2024 Sep 19;28(18):e70079. doi: 10.1111/jcmm.70079 (PMC11412914; doi:10.1111/jcmm.70079)
Supplement: Supplementary file 2 — Material S2. [file JCMM-28-e70079-s002.zip › 7.GSEA analysis/3.DBNDD1/KEGG_NEUROACTIVE_LIGAND_RECEPTOR_INTERACTION.html]

Details for gene set KEGG\_NEUROACTIVE\_LIGAND\_RECEPTOR\_INTERACTION[GSEA]

|  || Dataset | DBNDD1.group.cls#h\_versus\_l |
| Phenotype | group.cls#h\_versus\_l |
| Upregulated in class | h |
| GeneSet | KEGG\_NEUROACTIVE\_LIGAND\_RECEPTOR\_INTERACTION |
| Enrichment Score (ES) | 0.2733068 |
| Normalized Enrichment Score (NES) | 0.99309033 |
| Nominal p-value | 0.45746693 |
| FDR q-value | 1.0 |
| FWER p-Value | 1.0 |
Table: GSEA Results Summary

  

Fig 1: Enrichment plot: KEGG\_NEUROACTIVE\_LIGAND\_RECEPTOR\_INTERACTION      
 Profile of the Running ES Score & Positions of GeneSet Members on the Rank Ordered List

  

| SYMBOL | TITLE | RANK IN GENE LIST | RANK METRIC SCORE | RUNNING ES | CORE ENRICHMENT || 1 | GRID1 | na | 55 | 0.668 | 0.0134 | Yes |
| 2 | GNRHR | na | 61 | 0.656 | 0.0280 | Yes |
| 3 | CNR1 | na | 257 | 0.523 | 0.0342 | Yes |
| 4 | TRPV1 | na | 269 | 0.518 | 0.0455 | Yes |
| 5 | CHRNB2 | na | 305 | 0.507 | 0.0559 | Yes |
| 6 | DRD5 | na | 310 | 0.504 | 0.0671 | Yes |
| 7 | GHR | na | 396 | 0.477 | 0.0754 | Yes |
| 8 | CHRNA4 | na | 447 | 0.465 | 0.0844 | Yes |
| 9 | CHRNA1 | na | 490 | 0.456 | 0.0934 | Yes |
| 10 | GALR2 | na | 653 | 0.420 | 0.0982 | Yes |
| 11 | HCRTR2 | na | 695 | 0.415 | 0.1063 | Yes |
| 12 | GABRR2 | na | 754 | 0.407 | 0.1138 | Yes |
| 13 | CHRM5 | na | 780 | 0.405 | 0.1222 | Yes |
| 14 | GABRG3 | na | 788 | 0.404 | 0.1311 | Yes |
| 15 | GLP1R | na | 864 | 0.398 | 0.1378 | Yes |
| 16 | P2RX6 | na | 895 | 0.394 | 0.1458 | Yes |
| 17 | OPRM1 | na | 996 | 0.383 | 0.1516 | Yes |
| 18 | HTR6 | na | 1188 | 0.363 | 0.1542 | Yes |
| 19 | GRIK2 | na | 1265 | 0.356 | 0.1600 | Yes |
| 20 | ADRA1D | na | 1280 | 0.355 | 0.1675 | Yes |
| 21 | MTNR1A | na | 1359 | 0.348 | 0.1731 | Yes |
| 22 | NTSR1 | na | 1535 | 0.333 | 0.1755 | Yes |
| 23 | CHRNB4 | na | 1693 | 0.321 | 0.1782 | Yes |
| 24 | ADORA1 | na | 1718 | 0.319 | 0.1847 | Yes |
| 25 | TAAR8 | na | 2016 | 0.299 | 0.1828 | Yes |
| 26 | GABRA2 | na | 2020 | 0.298 | 0.1894 | Yes |
| 27 | TSHR | na | 2024 | 0.298 | 0.1960 | Yes |
| 28 | ADORA3 | na | 2064 | 0.295 | 0.2015 | Yes |
| 29 | HTR2C | na | 2350 | 0.279 | 0.1995 | Yes |
| 30 | GRM5 | na | 2394 | 0.277 | 0.2045 | Yes |
| 31 | HTR1A | na | 2478 | 0.273 | 0.2082 | Yes |
| 32 | THRB | na | 2499 | 0.272 | 0.2137 | Yes |
| 33 | GH1 | na | 2516 | 0.272 | 0.2194 | Yes |
| 34 | CHRNA2 | na | 2558 | 0.270 | 0.2243 | Yes |
| 35 | NPY2R | na | 2859 | 0.257 | 0.2213 | Yes |
| 36 | MC1R | na | 2945 | 0.254 | 0.2246 | Yes |
| 37 | VIPR2 | na | 3062 | 0.249 | 0.2268 | Yes |
| 38 | OPRK1 | na | 3250 | 0.241 | 0.2268 | Yes |
| 39 | CGA | na | 3378 | 0.236 | 0.2285 | Yes |
| 40 | NMUR2 | na | 3644 | 0.226 | 0.2258 | Yes |
| 41 | MC4R | na | 3739 | 0.222 | 0.2281 | Yes |
| 42 | GRM6 | na | 3839 | 0.219 | 0.2302 | Yes |
| 43 | SCTR | na | 3908 | 0.216 | 0.2330 | Yes |
| 44 | TACR3 | na | 4039 | 0.212 | 0.2341 | Yes |
| 45 | GPR50 | na | 4055 | 0.212 | 0.2384 | Yes |
| 46 | BDKRB2 | na | 4109 | 0.210 | 0.2416 | Yes |
| 47 | GABRA3 | na | 4240 | 0.205 | 0.2424 | Yes |
| 48 | GRM4 | na | 4259 | 0.204 | 0.2465 | Yes |
| 49 | NPY4R | na | 4453 | 0.198 | 0.2454 | Yes |
| 50 | TRHR | na | 4483 | 0.198 | 0.2490 | Yes |
| 51 | GABRG1 | na | 4727 | 0.189 | 0.2462 | Yes |
| 52 | RXFP2 | na | 4828 | 0.185 | 0.2474 | Yes |
| 53 | CCKAR | na | 4896 | 0.184 | 0.2496 | Yes |
| 54 | LPAR3 | na | 4933 | 0.182 | 0.2527 | Yes |
| 55 | GABRB3 | na | 4934 | 0.182 | 0.2568 | Yes |
| 56 | SSTR1 | na | 4943 | 0.182 | 0.2606 | Yes |
| 57 | F2R | na | 5139 | 0.176 | 0.2589 | Yes |
| 58 | PTGER3 | na | 5277 | 0.171 | 0.2588 | Yes |
| 59 | DRD3 | na | 5305 | 0.170 | 0.2618 | Yes |
| 60 | HRH3 | na | 5371 | 0.168 | 0.2637 | Yes |
| 61 | HTR5A | na | 5482 | 0.165 | 0.2643 | Yes |
| 62 | LPAR1 | na | 5576 | 0.162 | 0.2652 | Yes |
| 63 | ADRA2B | na | 5678 | 0.159 | 0.2658 | Yes |
| 64 | ADRA2A | na | 5752 | 0.157 | 0.2673 | Yes |
| 65 | P2RX5 | na | 5776 | 0.156 | 0.2701 | Yes |
| 66 | GRIK1 | na | 5932 | 0.152 | 0.2690 | Yes |
| 67 | LEP | na | 6081 | 0.148 | 0.2681 | Yes |
| 68 | RXFP1 | na | 6182 | 0.146 | 0.2684 | Yes |
| 69 | S1PR3 | na | 6202 | 0.145 | 0.2712 | Yes |
| 70 | AGTR2 | na | 6275 | 0.143 | 0.2723 | Yes |
| 71 | CALCRL | na | 6350 | 0.141 | 0.2733 | Yes |
| 72 | LTB4R | na | 7234 | 0.119 | 0.2504 | No |
| 73 | NPFFR2 | na | 7540 | 0.113 | 0.2441 | No |
| 74 | GRIN3A | na | 7721 | 0.109 | 0.2413 | No |
| 75 | SSTR3 | na | 7791 | 0.108 | 0.2417 | No |
| 76 | PTGER2 | na | 7863 | 0.106 | 0.2420 | No |
| 77 | GRIA2 | na | 7872 | 0.106 | 0.2442 | No |
| 78 | P2RY2 | na | 8305 | 0.098 | 0.2339 | No |
| 79 | LPAR6 | na | 8780 | 0.089 | 0.2221 | No |
| 80 | MC3R | na | 8992 | 0.086 | 0.2179 | No |
| 81 | GALR1 | na | 9120 | 0.084 | 0.2161 | No |
| 82 | CHRNA10 | na | 9123 | 0.084 | 0.2179 | No |
| 83 | NPFFR1 | na | 9267 | 0.082 | 0.2156 | No |
| 84 | GIPR | na | 9448 | 0.079 | 0.2122 | No |
| 85 | GLRA1 | na | 9644 | 0.076 | 0.2082 | No |
| 86 | S1PR5 | na | 9857 | 0.073 | 0.2037 | No |
| 87 | GLRB | na | 9931 | 0.072 | 0.2032 | No |
| 88 | MLNR | na | 9945 | 0.072 | 0.2045 | No |
| 89 | NTSR2 | na | 10070 | 0.070 | 0.2025 | No |
| 90 | PTGER1 | na | 10075 | 0.070 | 0.2039 | No |
| 91 | GPR156 | na | 10090 | 0.070 | 0.2051 | No |
| 92 | GCGR | na | 10447 | 0.065 | 0.1962 | No |
| 93 | PTGIR | na | 10527 | 0.064 | 0.1954 | No |
| 94 | GLRA3 | na | 10579 | 0.064 | 0.1953 | No |
| 95 | GABRA1 | na | 10580 | 0.064 | 0.1968 | No |
| 96 | P2RY13 | na | 10696 | 0.062 | 0.1948 | No |
| 97 | PTAFR | na | 10719 | 0.062 | 0.1956 | No |
| 98 | GRIN2A | na | 10969 | 0.059 | 0.1897 | No |
| 99 | ADRB2 | na | 11057 | 0.058 | 0.1884 | No |
| 100 | HTR4 | na | 11136 | 0.057 | 0.1874 | No |
| 101 | PLG | na | 11337 | 0.054 | 0.1829 | No |
| 102 | LTB4R2 | na | 11514 | 0.052 | 0.1789 | No |
| 103 | TAAR6 | na | 11598 | 0.051 | 0.1777 | No |
| 104 | TSPO | na | 11624 | 0.051 | 0.1781 | No |
| 105 | GRIN2C | na | 11804 | 0.049 | 0.1740 | No |
| 106 | TAAR1 | na | 11882 | 0.048 | 0.1728 | No |
| 107 | S1PR1 | na | 11978 | 0.047 | 0.1711 | No |
| 108 | CHRM2 | na | 12324 | 0.044 | 0.1621 | No |
| 109 | TSHB | na | 12345 | 0.044 | 0.1625 | No |
| 110 | PTH2R | na | 12431 | 0.043 | 0.1610 | No |
| 111 | CYSLTR2 | na | 12473 | 0.043 | 0.1608 | No |
| 112 | F2RL3 | na | 12626 | 0.042 | 0.1573 | No |
| 113 | HTR1B | na | 12708 | 0.041 | 0.1559 | No |
| 114 | GH2 | na | 12931 | 0.039 | 0.1503 | No |
| 115 | GABRR1 | na | 13242 | 0.036 | 0.1421 | No |
| 116 | DRD4 | na | 13629 | 0.033 | 0.1317 | No |
| 117 | HTR2B | na | 13672 | 0.033 | 0.1312 | No |
| 118 | TAAR9 | na | 13965 | 0.030 | 0.1234 | No |
| 119 | TAAR2 | na | 14005 | 0.030 | 0.1229 | No |
| 120 | OPRD1 | na | 14044 | 0.030 | 0.1225 | No |
| 121 | TAAR5 | na | 14382 | 0.027 | 0.1133 | No |
| 122 | GABRA6 | na | 14439 | 0.027 | 0.1123 | No |
| 123 | MAS1 | na | 14526 | 0.026 | 0.1104 | No |
| 124 | CHRM4 | na | 14597 | 0.026 | 0.1090 | No |
| 125 | CSH1 | na | 14842 | 0.024 | 0.1024 | No |
| 126 | C3AR1 | na | 15507 | 0.019 | 0.0836 | No |
| 127 | BRS3 | na | 15631 | 0.018 | 0.0804 | No |
| 128 | HTR1F | na | 15666 | 0.018 | 0.0798 | No |
| 129 | EDNRB | na | 15833 | 0.017 | 0.0754 | No |
| 130 | PARD3 | na | 16208 | 0.014 | 0.0649 | No |
| 131 | HCRTR1 | na | 16391 | 0.013 | 0.0599 | No |
| 132 | PRLHR | na | 17429 | 0.007 | 0.0300 | No |
| 133 | CNR2 | na | 17678 | 0.005 | 0.0229 | No |
| 134 | LPAR4 | na | 17835 | 0.004 | 0.0184 | No |
| 135 | LHB | na | 18008 | 0.003 | 0.0135 | No |
| 136 | PRL | na | 18062 | 0.002 | 0.0120 | No |
| 137 | NPY1R | na | 18468 | -0.001 | 0.0003 | No |
| 138 | MC5R | na | 18710 | -0.002 | -0.0067 | No |
| 139 | GABRG2 | na | 18819 | -0.003 | -0.0097 | No |
| 140 | SSTR4 | na | 18937 | -0.004 | -0.0130 | No |
| 141 | TBXA2R | na | 19010 | -0.005 | -0.0150 | No |
| 142 | GABRA5 | na | 19029 | -0.005 | -0.0154 | No |
| 143 | HTR1E | na | 19653 | -0.010 | -0.0333 | No |
| 144 | TACR2 | na | 19814 | -0.011 | -0.0377 | No |
| 145 | F2RL1 | na | 19857 | -0.012 | -0.0386 | No |
| 146 | CYSLTR1 | na | 19884 | -0.012 | -0.0391 | No |
| 147 | GRIN2D | na | 20127 | -0.014 | -0.0458 | No |
| 148 | THRA | na | 20666 | -0.019 | -0.0610 | No |
| 149 | GHRHR | na | 20706 | -0.019 | -0.0617 | No |
| 150 | HTR7 | na | 20721 | -0.019 | -0.0617 | No |
| 151 | NPBWR2 | na | 20761 | -0.020 | -0.0624 | No |
| 152 | SSTR5 | na | 21251 | -0.024 | -0.0760 | No |
| 153 | GZMA | na | 21345 | -0.025 | -0.0782 | No |
| 154 | C5AR1 | na | 21370 | -0.026 | -0.0783 | No |
| 155 | CHRNA9 | na | 21573 | -0.028 | -0.0835 | No |
| 156 | HRH2 | na | 21755 | -0.029 | -0.0881 | No |
| 157 | TACR1 | na | 22091 | -0.033 | -0.0971 | No |
| 158 | GALR3 | na | 22162 | -0.034 | -0.0984 | No |
| 159 | GABRA4 | na | 22281 | -0.035 | -0.1010 | No |
| 160 | GRM7 | na | 22342 | -0.036 | -0.1019 | No |
| 161 | FPR2 | na | 22419 | -0.036 | -0.1033 | No |
| 162 | ADRB1 | na | 22618 | -0.039 | -0.1082 | No |
| 163 | CHRNE | na | 22783 | -0.041 | -0.1121 | No |
| 164 | CHRNG | na | 22800 | -0.041 | -0.1116 | No |
| 165 | GABRQ | na | 22855 | -0.041 | -0.1122 | No |
| 166 | NMBR | na | 23037 | -0.043 | -0.1165 | No |
| 167 | CHRNB1 | na | 23104 | -0.044 | -0.1174 | No |
| 168 | CHRNA5 | na | 23195 | -0.046 | -0.1190 | No |
| 169 | CHRNB3 | na | 23551 | -0.050 | -0.1282 | No |
| 170 | F2 | na | 23840 | -0.054 | -0.1353 | No |
| 171 | FPR3 | na | 24027 | -0.057 | -0.1395 | No |
| 172 | OPRL1 | na | 24116 | -0.058 | -0.1407 | No |
| 173 | DRD1 | na | 24200 | -0.059 | -0.1418 | No |
| 174 | NPBWR1 | na | 24334 | -0.061 | -0.1443 | No |
| 175 | UTS2R | na | 24414 | -0.062 | -0.1452 | No |
| 176 | CTSG | na | 24437 | -0.062 | -0.1444 | No |
| 177 | P2RX2 | na | 24482 | -0.063 | -0.1443 | No |
| 178 | HRH1 | na | 24753 | -0.067 | -0.1506 | No |
| 179 | P2RY10 | na | 24781 | -0.068 | -0.1499 | No |
| 180 | P2RY14 | na | 24933 | -0.070 | -0.1527 | No |
| 181 | CHRNA7 | na | 25117 | -0.072 | -0.1564 | No |
| 182 | MCHR1 | na | 25196 | -0.074 | -0.1570 | No |
| 183 | GRM3 | na | 25335 | -0.076 | -0.1593 | No |
| 184 | P2RX3 | na | 25340 | -0.076 | -0.1577 | No |
| 185 | AVPR2 | na | 25376 | -0.077 | -0.1570 | No |
| 186 | GRPR | na | 25523 | -0.079 | -0.1594 | No |
| 187 | GHSR | na | 25597 | -0.080 | -0.1597 | No |
| 188 | CRHR1 | na | 25671 | -0.081 | -0.1600 | No |
| 189 | P2RX1 | na | 25792 | -0.083 | -0.1616 | No |
| 190 | P2RX4 | na | 25935 | -0.086 | -0.1638 | No |
| 191 | GRM8 | na | 26089 | -0.089 | -0.1663 | No |
| 192 | FSHB | na | 26117 | -0.090 | -0.1650 | No |
| 193 | FSHR | na | 26147 | -0.091 | -0.1638 | No |
| 194 | CHRNA3 | na | 26321 | -0.094 | -0.1667 | No |
| 195 | ADORA2B | na | 26330 | -0.094 | -0.1648 | No |
| 196 | BDKRB1 | na | 26699 | -0.102 | -0.1732 | No |
| 197 | P2RY1 | na | 26719 | -0.102 | -0.1715 | No |
| 198 | ADORA2A | na | 26738 | -0.102 | -0.1697 | No |
| 199 | GABBR1 | na | 26807 | -0.104 | -0.1694 | No |
| 200 | GRIN2B | na | 26883 | -0.105 | -0.1692 | No |
| 201 | GABRB1 | na | 26974 | -0.107 | -0.1694 | No |
| 202 | NR3C1 | na | 26975 | -0.107 | -0.1670 | No |
| 203 | S1PR2 | na | 27102 | -0.110 | -0.1681 | No |
| 204 | HTR1D | na | 27151 | -0.111 | -0.1670 | No |
| 205 | GRIN1 | na | 27251 | -0.114 | -0.1674 | No |
| 206 | GRID2 | na | 27375 | -0.116 | -0.1683 | No |
| 207 | ADRA1A | na | 27600 | -0.121 | -0.1721 | No |
| 208 | GABBR2 | na | 27736 | -0.125 | -0.1732 | No |
| 209 | AVPR1B | na | 27779 | -0.126 | -0.1716 | No |
| 210 | GLP2R | na | 27806 | -0.126 | -0.1695 | No |
| 211 | PRSS3 | na | 27833 | -0.127 | -0.1674 | No |
| 212 | CCKBR | na | 27988 | -0.131 | -0.1689 | No |
| 213 | LPAR2 | na | 28055 | -0.132 | -0.1679 | No |
| 214 | ADRA2C | na | 28107 | -0.133 | -0.1663 | No |
| 215 | DRD2 | na | 28171 | -0.135 | -0.1651 | No |
| 216 | PTH1R | na | 28349 | -0.139 | -0.1671 | No |
| 217 | CHRND | na | 28356 | -0.139 | -0.1642 | No |
| 218 | GRIK5 | na | 28503 | -0.143 | -0.1652 | No |
| 219 | GPR35 | na | 28672 | -0.148 | -0.1667 | No |
| 220 | S1PR4 | na | 28981 | -0.156 | -0.1722 | No |
| 221 | LEPR | na | 29224 | -0.163 | -0.1755 | No |
| 222 | P2RY6 | na | 29321 | -0.167 | -0.1745 | No |
| 223 | HTR2A | na | 29589 | -0.175 | -0.1783 | No |
| 224 | GRM1 | na | 30327 | -0.199 | -0.1953 | No |
| 225 | AVPR1A | na | 30657 | -0.209 | -0.2001 | No |
| 226 | GRIA3 | na | 30739 | -0.212 | -0.1977 | No |
| 227 | ADRB3 | na | 30873 | -0.216 | -0.1967 | No |
| 228 | CHRM1 | na | 31045 | -0.222 | -0.1966 | No |
| 229 | NPY5R | na | 31171 | -0.227 | -0.1952 | No |
| 230 | FPR1 | na | 31173 | -0.227 | -0.1901 | No |
| 231 | P2RY4 | na | 31189 | -0.228 | -0.1854 | No |
| 232 | OXTR | na | 31229 | -0.230 | -0.1814 | No |
| 233 | ADCYAP1R1 | na | 31255 | -0.231 | -0.1769 | No |
| 234 | AGTR1 | na | 31373 | -0.235 | -0.1750 | No |
| 235 | GABRB2 | na | 31563 | -0.243 | -0.1750 | No |
| 236 | VIPR1 | na | 31625 | -0.246 | -0.1713 | No |
| 237 | GRIA4 | na | 31687 | -0.248 | -0.1675 | No |
| 238 | CHRM3 | na | 31862 | -0.257 | -0.1667 | No |
| 239 | PTGDR | na | 31882 | -0.258 | -0.1615 | No |
| 240 | GRIN3B | na | 31913 | -0.260 | -0.1565 | No |
| 241 | PTGFR | na | 31929 | -0.260 | -0.1511 | No |
| 242 | PTGER4 | na | 31982 | -0.263 | -0.1467 | No |
| 243 | NMUR1 | na | 31990 | -0.263 | -0.1410 | No |
| 244 | GRIA1 | na | 32166 | -0.273 | -0.1399 | No |
| 245 | ADRA1B | na | 32178 | -0.275 | -0.1341 | No |
| 246 | CHRNA6 | na | 32200 | -0.276 | -0.1285 | No |
| 247 | HRH4 | na | 32376 | -0.286 | -0.1271 | No |
| 248 | GABRE | na | 32515 | -0.295 | -0.1245 | No |
| 249 | P2RX7 | na | 32561 | -0.298 | -0.1191 | No |
| 250 | GABRP | na | 32714 | -0.307 | -0.1166 | No |
| 251 | LHCGR | na | 32721 | -0.307 | -0.1098 | No |
| 252 | GRIK3 | na | 32745 | -0.309 | -0.1036 | No |
| 253 | GABRD | na | 32760 | -0.311 | -0.0970 | No |
| 254 | CRHR2 | na | 32972 | -0.327 | -0.0958 | No |
| 255 | APLNR | na | 33241 | -0.347 | -0.0957 | No |
| 256 | CALCR | na | 33249 | -0.349 | -0.0881 | No |
| 257 | P2RY8 | na | 33298 | -0.353 | -0.0815 | No |
| 258 | GRIK4 | na | 33329 | -0.356 | -0.0744 | No |
| 259 | KISS1R | na | 33375 | -0.359 | -0.0676 | No |
| 260 | PRLR | na | 33449 | -0.366 | -0.0615 | No |
| 261 | SSTR2 | na | 33482 | -0.369 | -0.0542 | No |
| 262 | MTNR1B | na | 33491 | -0.370 | -0.0461 | No |
| 263 | GPR83 | na | 33747 | -0.397 | -0.0446 | No |
| 264 | F2RL2 | na | 33757 | -0.399 | -0.0359 | No |
| 265 | MC2R | na | 33768 | -0.400 | -0.0272 | No |
| 266 | GLRA2 | na | 33961 | -0.424 | -0.0232 | No |
| 267 | GRM2 | na | 33962 | -0.424 | -0.0136 | No |
| 268 | PRSS1 | na | 34218 | -0.467 | -0.0105 | No |
| 269 | MCHR2 | na | 34517 | -0.560 | -0.0066 | No |
| 270 | EDNRA | na | 34526 | -0.565 | 0.0059 | No |
Table: GSEA details [plain text format]

  

Fig 2: KEGG\_NEUROACTIVE\_LIGAND\_RECEPTOR\_INTERACTION      
 Blue-Pink O' Gram in the Space of the Analyzed GeneSet

  

Fig 3: KEGG\_NEUROACTIVE\_LIGAND\_RECEPTOR\_INTERACTION: Random ES distribution      
 Gene set null distribution of ES for **KEGG\_NEUROACTIVE\_LIGAND\_RECEPTOR\_INTERACTION**
